# Laser absorption spectroscopy measurements of different pulmonary oxygen gas concentrations in transmittance and remittance geometry: phantom study

**DOI:** 10.1117/1.JBO.28.11.115003

**Published:** 2023-11-30

**Authors:** Andrea Pacheco, Jean Matias, Konstantin Grygoryev, Martin Hansson, Sara Bergsten, Stefan Andersson-Engels

**Affiliations:** a Tyndall National Institute, Biophotonics@Tyndall, IPIC, Cork, Ireland; b University College Cork, Department of Physics, Cork, Ireland; c Neola Medical AB, Lund, Sweden

**Keywords:** computational model of a thorax, light transport, preterm infants, tuneable diode laser absorption spectroscopy

## Abstract

**Significance:**

The gas in scattering media absorption spectroscopy (GASMAS) technique has the potential for continuous, clinical monitoring of preterm infant lung function, removing the need for X-ray diagnosis and reliance on indirect and relatively slow measurement of blood oxygenation.

**Aim:**

We aim to determine the optimal source–detector configuration for reliable pathlength calculation and to estimate the oxygen gas concentration inside the lung cavities filled with humidified gas with four different oxygen gas concentrations ranging between 21% and 100%.

**Approach:**

Anthropomorphic optical phantoms of neonatal thorax with two different geometries were used to acquire GASMAS signals, for 30 source–detector configurations in transmittance and remittance geometry of phantoms in two sizes.

**Results:**

The results show that an internal light administration is more likely to provide a high GASMAS signal-to-noise ratio (SNR). In general, better SNRs were obtained with the smaller set of phantoms. The values of pathlength and $O_{2}$ concentrations calculated with signals from the phantoms with optical properties at 820 nm exhibit higher variations than signals from the phantoms with optical properties at 764 nm.

**Conclusion:**

Our study shows that, by moving the source and detector over the thorax, most of the lung volumes can potentially be assessed using the GASMAS technique.

## 1 Introduction

Approximately 1 million babies die every year due to complications of preterm birth.^1^ The lungs of neonates born before 37 completed weeks of pregnancy may suffer from structural and biochemical immaturity, which together with surfactant deficiency (lack of the phospholipid mixture that maintains alveolar stability) and incomplete vascularization cause respiratory distress syndrome (RDS).^2^ Despite the efforts to mitigate lung malfunction in neonates, RDS is still one of the major causes of mortality and morbidity among infants.^3^ Consequently, there is a compelling need to improve diagnostic and treatment tools for lung disease in neonates. The prevailing methods for diagnosis and monitoring of treatment response are pulse oximetry, blood gas analysis, chest radiography, and computer tomography (CT) scanning. The latter techniques use ionizing radiation projected onto the torso of the neonate, and repeated exposure to it, in particular, represents a risk to the neonate.^4^ Oxygen ($O_{2}$) administration is one of the mechanisms used in the intensive care unit to aid neonates with respiratory dysfunction; in this case, the delivered $O_{2}$ concentration varies between 30% and 95% depending of the need of the patient and is measured by a gas analyzer placed near the infant’s mouth.^5^

Gas in scattering media absorption spectroscopy (GASMAS) is a tunable diode laser spectroscopic (TDLAS) technique developed for nonintrusive detection and monitoring of free gas enclosed inside turbid media without extraction.^6^ In the clinic, GASMAS could potentially complement and, in some cases, even replace existing modalities, including radiography, CT, magnetic resonance imaging, and ultrasound. Feasibility studies have shown the potential of GASMAS to measure molecular oxygen ($O_{2}$) gas and water vapor ($H_{2}O$) in the lungs of neonates.^7^^–^^9^ Research efforts toward its clinical translation into neonatal respiratory health care have included animal studies,^10^ computational modeling,^11^ and phantom measurements.^12^^–^^14^ One of the remaining challenges is to better understand what source–detector configuration to use to acquire signals of the highest quality and stability. Several studies have been conducted to address this challenge.

Svanberg et al. conducted GASMAS studies in five mechanically ventilated piglets, which were subject to stepwise increased and decreased fractions of inspired $O_{2}$ (in concentrations between 20% and 97%), atelectasis (partial or total lung collapse), and pneumothorax (air leakage between the lung and the chest wall). Light from a dual source (764 and 820 nm) was delivered externally on the skin or internally with a source inside the oesophagus, and in both cases, the detector was placed in direct contact with the skin. The results showed that $H_{2}O$ vapor was only detectable with internal illumination, and specific light absorption patterns were identified in response to atelectasis and pneumothorax.^10^

Liao et al. simulated light transport at 764 nm for different source–detector pairs that were placed over the chest of a computational model of neonatal thorax. The authors concluded that measurement geometries with the probes placed in front and in the region under the armpits are favorable to obtaining good gas absorption signals.^9^

With the aim to better understand the advantages and challenges of translating GASMAS in neonatal respiratory healthcare, different optical phantoms have been made. Larsson et al. developed a three-dimensional nylon phantom, which consisted of four hollowed compartments with the boundary structure of skin, subcutaneous fat, lung, and heart. Each of them was designed to be filled with liquid phantom matching the respective tissue optical properties at 764 and 820 nm wavelengths. Initially, GASMAS measurements were made with gas-filled pulmonary cavities without any additional absorbing or scattering material, and reliable $O_{2}$ and $H_{2}O$ vapor absorption signals were found for source–detector separations up to 5 cm.^11^ In a follow up publication, the phantom included a structure with optical properties inside the lung, and GASMAS measurements were done to compare the differences in signal, when the probes were placed in remittance (source and detector over the chest) and transmittance (internal light source and detectors over the chest) geometries. The authors concluded that the internal light administration resulted in a larger gas absorption and higher signal-to-noise ratio (SNR) compared with dermal light administration.^12^

In the same spirit, the Biophotonics@Tyndall team created a lung tissue model that mimicked the alveolar structure to demonstrate the potential of the GASMAS technique to measure changes in the inflated volume.^13^

To further address the issue of measurement configuration and reproducibility, we present, in this paper, the results from GASMAS measurements made with four solid anthropomorphic optical phantoms that resemble the thoracic organs of two neonates. In this study, we also include an internal source placed in the oesophagus. A total of 30 source–detector geometries with detectors placed in the front and the back of the thorax were studied using four different gas mixtures. The main objectives of this study were to identify the best configurations to obtain good absorption signals of $O_{2}$ and $H_{2}O$ vapor at 764 and 820 nm, respectively, and to study the differences in the GASMAS signal linked to changes in the $O_{2}$ concentration present inside the pulmonary cavities of the phantoms.

## 2 Methods

### 2.1 GASMAS Principle

The GASMAS technique is based on the difference between the absorption features of gases and solid-state matter. Gases present spectrally sharp absorption lines (∼0.001  nm width),^15^ enabling sensitive measurements of gas concentrations in the presence of a scattering solid-state media with much broader absorption features (at least ∼10  nm width).^16^ In a typical GASMAS measurement targeting to sense $O_{2}$ and $H_{2}O$ vapor content, a dual diffuse laser diode source illuminates the walls of the scattering object enclosing gas. Photons scattered from the medium reach a photodetector, and the absorption signal from the gas is identified. One of the lasers scans an absorption line of molecular oxygen ($O_{2}$) gas around 764 nm, and the second scans an absorption line of water vapor ($H_{2}O$) around 820 or 934 nm. The wavelengths are spectrally close, which allows for the assumption that the pathlength within the tissue for the light of both laser diodes is approximately the same.

The partial pressure of water vapor in air ($e_{w}^{\prime }$) at ambient temperature (T) and pressure (P) can be determined using the Arden Buck equation^17^ at 100% relative humidity (RH). Using the ideal gas law equation, the water vapor concentration can then be calculated. The corresponding absorption coefficient of the water vapor $\mu_{a}$ was then obtained, using the vapor concentration together with the tabulated extinction coefficient in the molecular absorption (HITRAN) database. At room temperature (293 K) and 820 nm, the peak $\mu_{a}$ of water vapor was $3.1\times 10^{-5}\;{\mathrm{cm}}^{-1}$.^18^ The absorption pathlength was then estimated by means of the Beer–Lambert law:^19^

$$I=I_{0}e^{-\mu_{a}l},$$ (1)

Where I is the intensity of light at 820 nm reaching the detector, $I_{0}$ is the intensity of the light source, $\mu_{a}$ (${\mathrm{cm}}^{-1}$) is the absorption coefficient of the water vapor, and l (cm) is the gas absorption pathlength. Subsequently, the calculated value of the pathlength is used as an input parameter in Eq. (1) to estimate the $O_{2}$ concentration, using the absorption signal of light at 764 nm.

### 2.2 GASMAS Systems

Two systems were used in this study: a gas calibration cell (Gasporox CellSpect $O_{2}$) produced by GASPOROX was included in the protocol to verify the values of $O_{2}$ concentration prior to the gas administration into the phantom and the Neola™ system produced by Neola Medical AB.

The Neola™ system had a dual source made with two distributed-feedback laser diodes (Nanoplus). The emitted light was centered at wavelengths of 763.8 nm (20.3 mW) and 820.0 nm (16.6 mW), respectively, for the two lasers. The TDLAS measurements using the individual lasers were time-multiplexed at a switching frequency of 279 Hz. During each measurement cycle, the wavelength of the emitted light was scanned over one absorption line of the corresponding gas by modulation of the injection current. The light from both lasers was coupled into an optical fiber. To acquire gas absorption signals in transmittance geometry, the fiber was introduced inside the phantom at the level of the trachea. For remittance geometry, a custom $10\times 10\;{\mathrm{mm}}^{2}$ light diffuser was coupled to the fiber to reduce the laser power density and simulate clinical use. The transmitted light was collected with a photodiode with a $10\times 10\;{\mathrm{mm}}^{2}$ photosensitive area (S3590, HAMAMATSU).

### 2.3 Anthropomorphic Phantoms

Two different thoracic anatomies were used in this study for phantom fabrication. The organ geometry was recovered from anonymized CT scans of two neonates, weighing 3.7 and 3.6 kg, respectively. The detailed manufacturing protocol used in this study has been described in a previous publication by the Biophotonics@Tyndall team.^14^ Briefly, each CT was segmented to distinguish between seven different tissue types and organs (skin, fat, muscle, bone, heart, trachea, and lung). Models of heart, lung, and muscle were 3D printed to create moulding casts to preserve the anatomic structure of the thorax. For each anatomical geometry, two phantoms were produced, one with optical properties matching 764 nm and the second with 820 nm (total of 4). The values of absorption and reduced scattering coefficients ($\mu_{a}$ and $\mu_{s}^{\prime }$) at 764 and 820 nm were specified according to the available literature (see Table 1). The two sets of phantoms were used to determine if tissue optical properties had a significant influence on GASMAS measurements.

**Table 1:** Optical properties (absorption $\mu_{a}$ and reduced scattering $\mu_{s}^{\prime }$ coefficients) at 764 and 820 nm assigned to the solid tissue phantoms in the thoracic models.

	764 nm		820 nm		
Tissue	$\mu_{a}$ (${\mathrm{cm}}^{-1}$)	$\mu_{s}^{\prime }$ (${\mathrm{cm}}^{-1}$)	$\mu_{a}$ (${\mathrm{cm}}^{-1}$)	$\mu_{s}^{\prime }$ (${\mathrm{cm}}^{-1}$)	Reference
Skin	0.03	24.8	0.03	22.8	16
Fat	0.13	13.9	0.07	13.2	16 and 20
Muscle	0.20	14.1	0.20	12.8	16
Bone	0.10	9.30	0.11	8.40	16
Heart	0.25	4.89	0.11	4.45	16

**Fig. 1:**
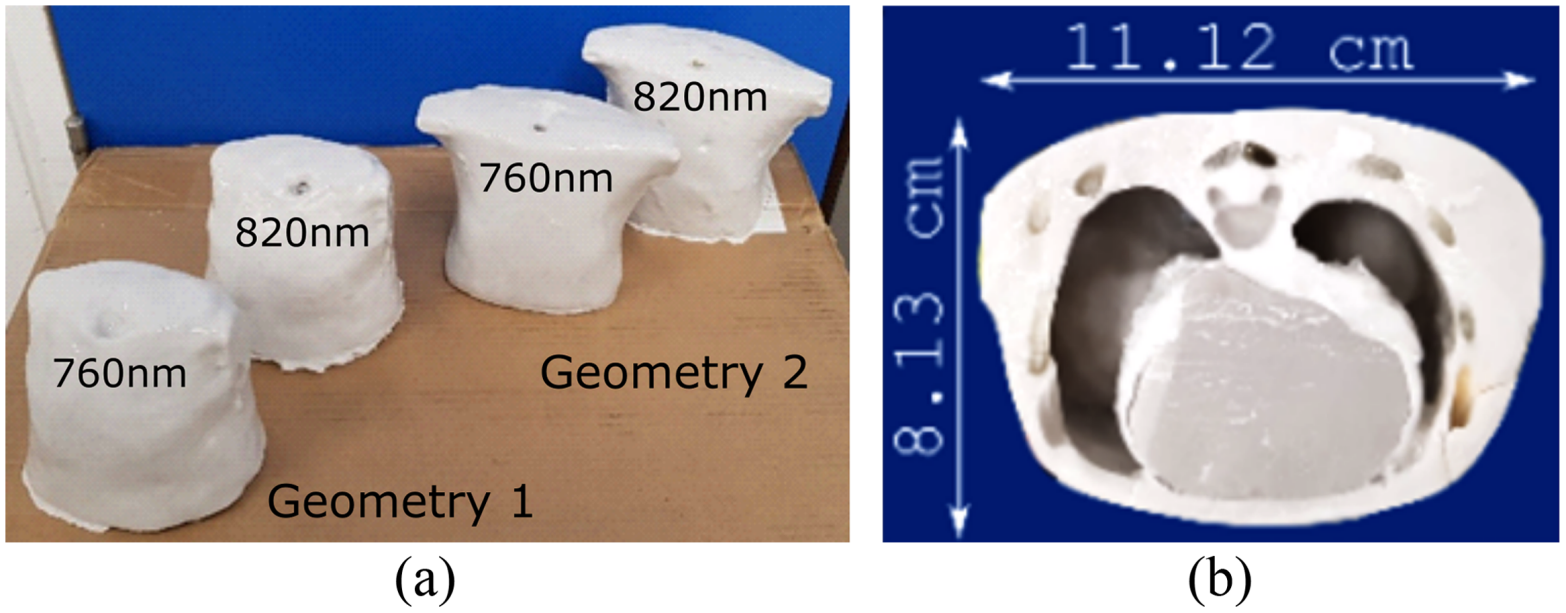
(a) Phantoms used to perform the GASMAS measurements in this study are illustrated. Geometries 1 and 2 correspond to the CT thoracic scans of 3.7 and 3.6 kg neonates, respectively. Phantoms labeled with 760 and 820 nm correspond with tissue optical properties at 760 and 820 nm, respectively. (b) Cross section through a phantom showing the position of the heart and its influence on the lung volume.

Each organ was modeled in the phantoms as optically homogeneous, and small structures usually present in tissue such as veins or collagen were not considered. All phantoms were made with empty pulmonary cavities that facilitated gas exchange through a 5 mm tracheal hole, which has two branches connected to the lungs, as can be seen in Fig. 1.

### 2.4 Experimental Set Up

**Fig. 2:**
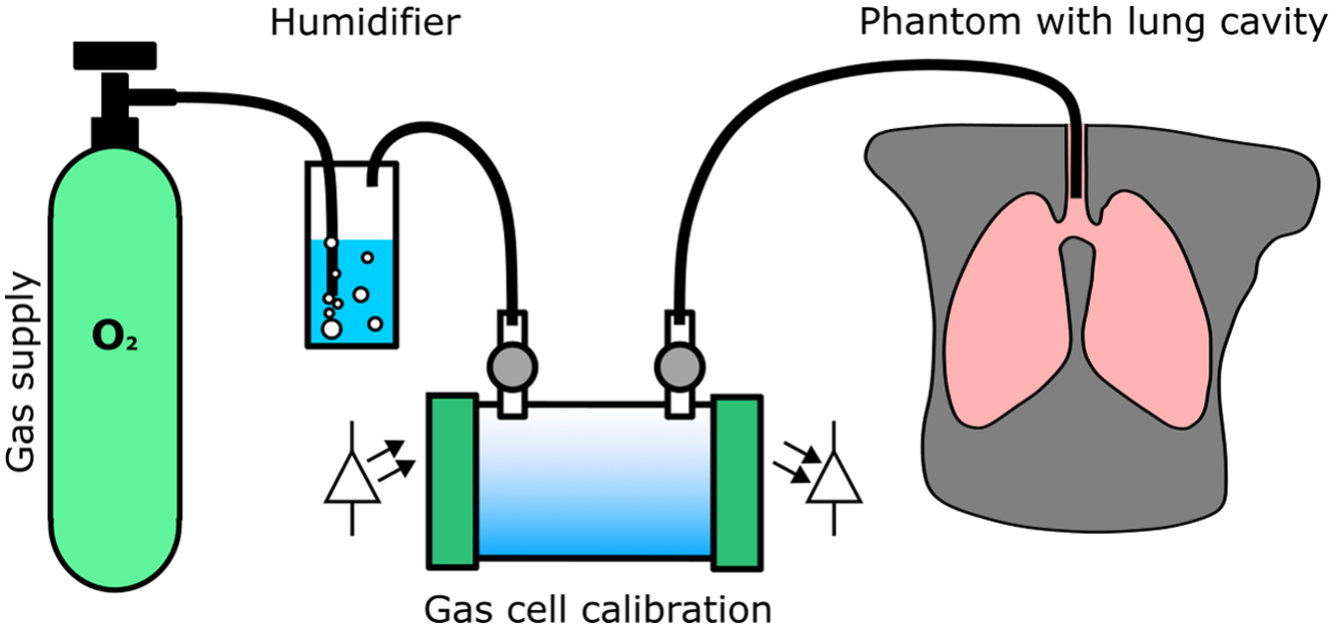
Diagram of the experimental protocol followed in this study. Gas with a specific $O_{2}$ concentration was humidified; then it was passed through a calibration cell to verify 100% RH and $O_{2}$ percentage prior to entering the pulmonary cavities of the phantom.

The diagram of the protocol used in this study is shown in Fig. 2. Gases with 21%, 30%, 50%, and 100% $O_{2}$ concentration were humidified and delivered inside the pulmonary cavities of each phantom. The range was selected to reduce the risk of a potentially low SNR preventing data acquisition. The humid gas was assessed using the GASPOROX calibration cell prior to its delivery into the phantom to verify the $O_{2}$ concentration. Neola™ source and detectors were fixed to the phantom to take the measurements for the remittance and transmittance geometries, as described in the following section.

### 2.5 Measurement Configurations

**Fig. 3:**
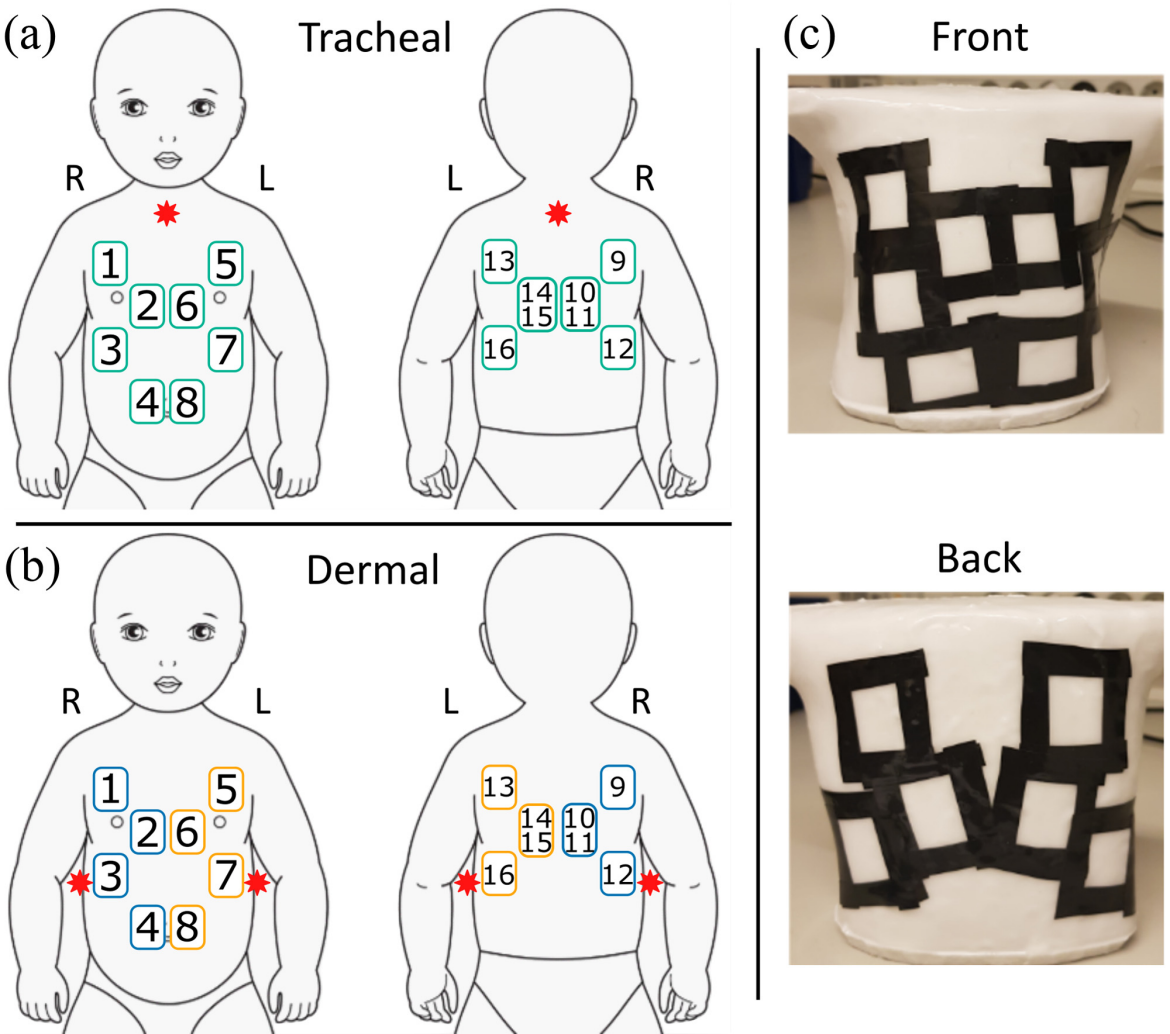
Diagrams show the source–detector configurations used to measure the gas content in the phantom’s lung in (a) transmittance and (b) remittance geometries. (c) The black mask placed on the phantoms to prevent stray light from the source reaching the detector. Blue and yellow marked detector locations correspond to right and left source positions, respectively.

Thirty source–detector configurations were tested in this study. Figure 3(a) shows the 15 source–detector pairs in transmittance geometry with the source placed internally in the trachea and the detector over the thorax. Figure 3(b) corresponds to the 16 remittance geometries with the source and detector placed over the phantom’s skin on the front and back on the left or right. The source was placed under the armpit on the right or left side of the phantom. The placement of the source and detectors was balanced and avoided detector saturation with light that did not pass through any gas as well as the absence of any optical signal due to strong tissue attenuation.^21^

The black mask shown in Fig. 3(c) was used in all measurements to prevent the detection of stray light (photons traveling from the source to the detector without interaction with the phantoms). The detector positions labeled with numbers 10 and 11 correspond to two consecutive measurements taken in the exact same position (as is the case of 14 and 15), which was made to evaluate the reproducibility of the measurements.

The measurement protocol of Fig. 2 was used to acquire GASMAS measurements with all four phantoms, conducting 21 repeated measurements reattaching the detectors each time for each sample point ($O_{2}$ and $H_{2}O$ vapor absorption signals at 764 and 820 nm, respectively; 30 different source–detector configurations; administrating gas with 21%, 30%, 50%, and 100% $O_{2}$ concentration).

GASMAS measures the average pathlength of light through the gas; therefore, this quantity will depend on the gas volume between the probes at constant RH, T, and ambient P. Because the gas was humidified and maintained at the same room conditions during the study, it was expected that the pathlength in all 30 source–detector configurations remained the same for each phantom pair with geometries 1 and 2, regardless the $O_{2}$ content in the gas. The pathlength for 21 samples in each source–detector configuration was calculated and averaged. Data points with calculated pathlengths that were either 0 m or >0.23  m were categorized as outliers.

## 3 Results and Discussion

**Fig. 4:**
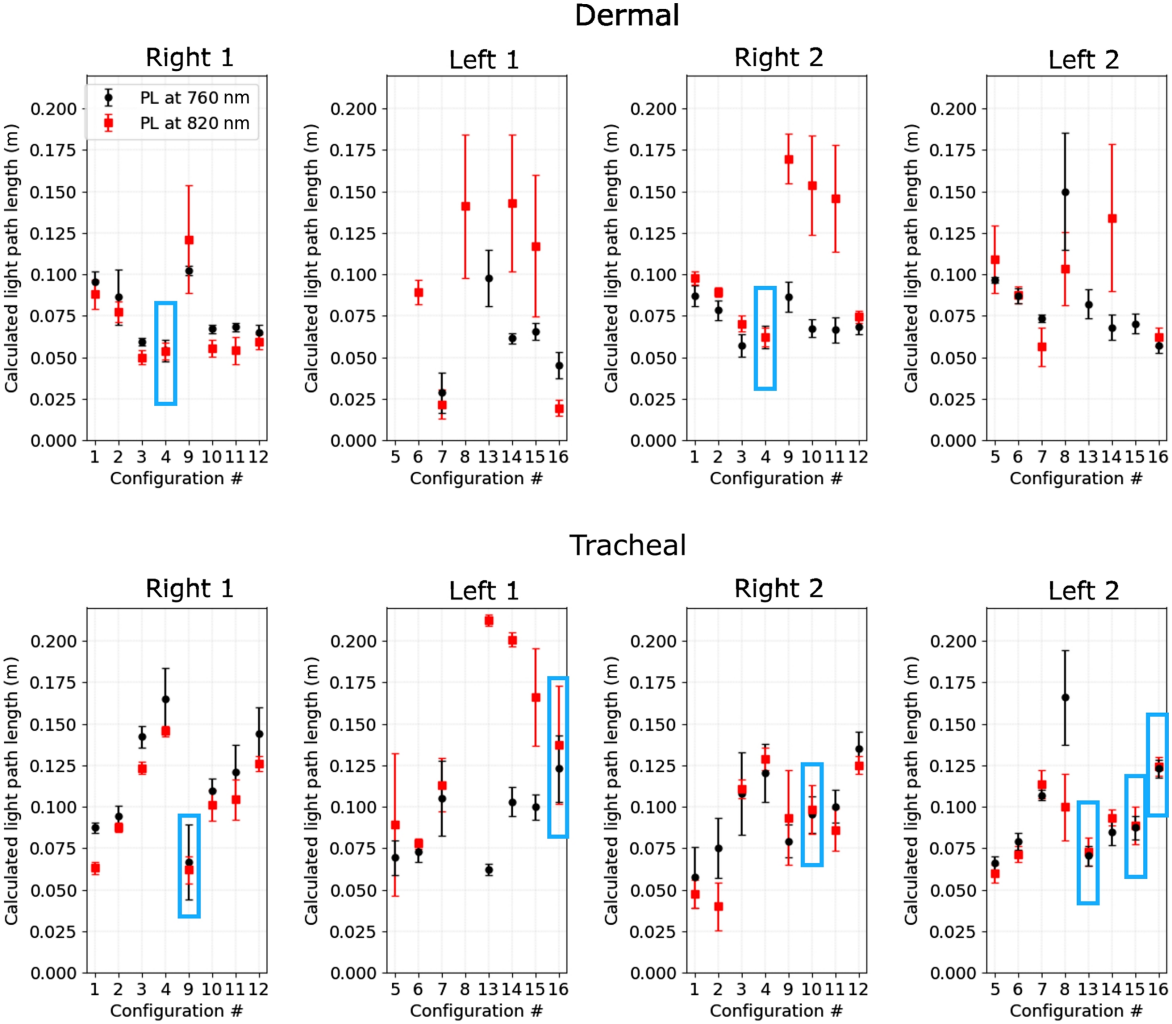
Calculated mean and standard deviation pathlength values for the both phantom geometries (1 and 2), optical properties (764 and 820 nm), and oxygen concentrations 21% and 30%, in remittance and transmittance geometries. Blue boxes indicate source–detector configurations with p>0.05 following the Kolmogorov–Smirnov t-test, indicating pathlengths that statistically cannot be differentiated. Right/left 1, geometry 1 and right/left 2, geometry 2.

Figure 4 shows the average calculated pathlengths for two phantom geometries and combined $O_{2}$ concentrations of 21% and 30% with light that was administrated internally (tracheal) and over the skin (dermal). The measurement configurations for which the pathlength overlaps the most for the two phantoms with the same geometry correspond to the source–detector combinations that may lead to the most reliable GASMAS signals. For example, “dermal” source–detector configuration #4 in both phantoms showed similar pathlengths for both wavelengths indicated by p>0.05 following a Kolmogorov–Smirnov t-test. In addition, from a qualitative perspective, configurations #1, 2, and 3 on the right side of the phantom have the potential for an accurate calculation of $O_{2}$ concentration as well.

The “tracheal” illumination configurations #9, 10, 13, 15, and 16 showed comparable pathlengths (p>0.05) for both wavelengths, although configuration #16 was the only overlap between two phantom geometries. From a qualitative perspective, only configurations #5, 6, and 7 of geometry 2 may be considered suitable for GASMAS measurement.

The locations of optimal source–detector positions and pathlength calculations were influenced by (i) the thickness of the thoracic wall, (ii) the significant difference of the heart volumes, and (iii) the natural tendency of the heart to be located to the left of the sternum (inset of Fig. 1). The volume of the heart in geometry 1 was 111 ml versus 56 ml in geometry 2. This resulted in (i) decreased volume or sampled air and (ii) strong tissue scattering and attenuation of light across the entire scanned spectrum, leading to a severe reduction of the SNR. For example, a low SNR is especially evident on left side of geometry 1 (Fig. 4, left 1) where the calculated pathlengths for 764 or 820 nm were either noticeably different, zero, or above the 200 mm threshold.

**Fig. 5:**
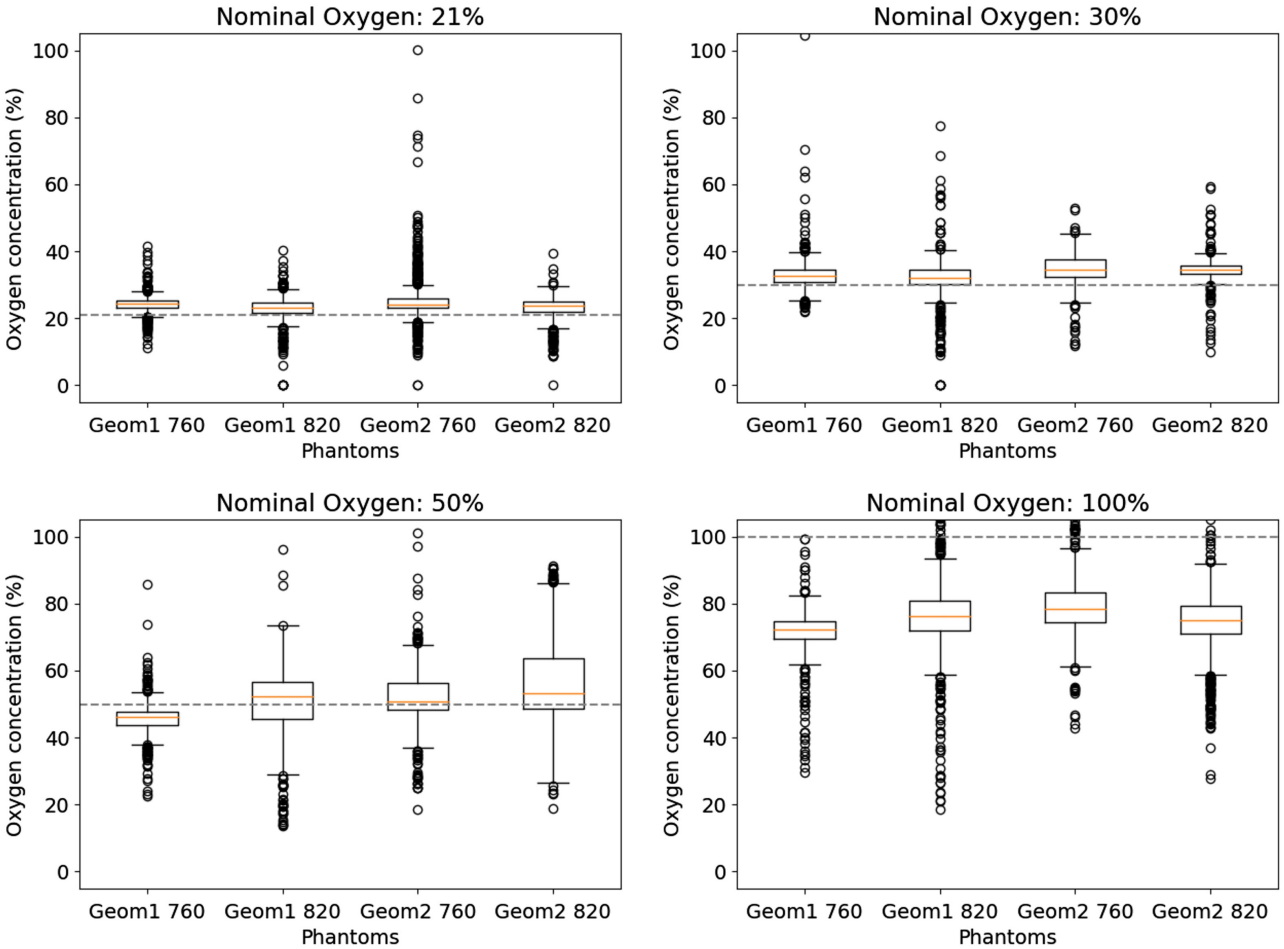
Plots of the values of $O_{2}$ concentration measured with the Neola™ system. The dotted lines correspond to the nominal values of $O_{2}$ content of the gas input in the phantom cavities.

Figure 5 shows box plots of the calculated $O_{2}$ concentrations taken with the full set of phantoms and all source–detector configurations. The red lines in each box correspond to the median value, and the black circles represent the outliers designated as discussed above. The nominal values of gas input for each measurement batch are represented by the dotted line. Ideally, the same gas concentration should be estimated for all GASMAS samples at a specific gas badge. However, the estimated concentrations differ from the nominal values of $O_{2}$ percentage, exhibiting a low overestimation in the cases of 21% and 30%, a larger standard deviation for 50%, and a notable underestimation with a high standard deviation for 100% $O_{2}$ supply.

The probable reason for the larger standard deviation and the underestimation is related to gas mixing within the lung cavities caused by insufficient purging. The 100% $O_{2}$ was potentially mixed with air at 21%, 30%, and 50% in the lung, and the calibration cell, being an easily purged simple geometry, indicated that the concentration was at the required 50% or 100%.

The phantom models used in this study lacked a structure to mimic the absorption of scattering due to the lung tissue itself. In the real clinical scenario, the GASMAS signals may be further attenuated given the optical properties of lung ($\mu_{a760}=0.5\;{\mathrm{cm}}^{-1}$, $\mu_{s760}^{\prime }=5.4\;{\mathrm{cm}}^{-1}$, $\mu_{a820}=0.7\;{\mathrm{cm}}^{-1}$, and $\mu_{s820}^{\prime }=4.9\;{\mathrm{cm}}^{-1}$).^17^ In such cases, the optimal positioning is even more critical.

The placement of the endotracheal light source results in more reliable measurements when detectors are placed on the back of the torso (configurations #9 to 16 in Fig. 4). The internal light administration improves the interaction of detected light with the gas inside the pulmonary cavities because, this geometry makes it necessary for the light to travel through the gas, before reaching the detectors over the torso. When the source and detector are placed in the remittance geometry, the light undergoes double attenuation by tissue surrounding the cavities. Preterm infants are often intubated for breathing or feeding proposes. In those cases, placing a light source in the trachea would be feasible. However, the main aim of GASMAS technology translated into respiratory healthcare is a noninvasive assessment of lung function, and the endotracheal light source should be used only if the infant is already intubated. The localization of a source optical fiber inside the respiratory or feeding tube could be prototyped and evaluated.

## 4 Conclusion

GASMAS bench top measurements were conducted with a set of solid anthropomorphic phantoms with geometries obtained from two different neonatal chest CT scans.

The results showed that the source–detector configurations have an observable effect on the calculated pathlengths of 764 and 820 nm wavelengths. The configurations that showed the smallest discrepancy have the highest probability of measuring oxygen concentration accurately and thus have the most clinical benefit. As, between one and three, dermal configurations showed low pathlengths discrepancies, this provides clinicians with a choice of probe placements to optimize monitoring.

The tracheal light administration showed a higher number of source–detector configurations, indicating that such arrangements would be preferable for accurate measurements of gas concentration. As such, intubated patients under extended observation could benefit from this arrangement because the endotracheal tubes can be easily integrated with an optical fiber to deliver the light into the lungs. Furthermore, such an arrangement has the potential to be used on adult patients, further expanding the clinical benefit of GASMAS lung function monitoring.

Finally, this study confirmed that GASMAS can be used to directly estimate the changes in oxygen concentration inside a diffuse media with optical properties of biological tissue, further highlighting the benefit of this technique in a clinical setting.

Overall, the results from this study may assist in optimizing the source–detector configurations to perform GASMAS measurements in the clinic, which could enable recordings of the oxygen levels in the lung. We have shown that, by changing the position of source and detector over the torso, most of the lung can be assessed.

## Data Availability

The data that support the findings of this study are openly available at the public repository Zenodo.org. DOI: 10.5281/zenodo.8282578.
